# Ghrelin inhibits LPS-induced release of IL-6 from mouse dopaminergic neurones

**DOI:** 10.1186/1742-2094-10-40

**Published:** 2013-03-19

**Authors:** Amy L Beynon, M Rowan Brown, Rhiannon Wright, Mark I Rees, I Martin Sheldon, Jeffrey S Davies

**Affiliations:** 1 Molecular Neuroscience, Swansea University, Swansea, SA28PP, UK; 2 Center for Nanohealth, Swansea University, Swansea, SA28PP, UK; 3 Innate Immunity, Institute of Life Sciences, Swansea University, Swansea, SA28PP, UK

**Keywords:** Ghrelin, Interleukin-6, Dopamine, Neurones, Lipopolysaccharide, Parkinson’s disease

## Abstract

**Background:**

Ghrelin is an orexigenic stomach hormone that acts centrally to increase mid-brain dopamine neurone activity, amplify dopamine signaling and protect against neurotoxin-induced dopamine cell death in the mouse *substantia nigra pars compacta* (SNpc). In addition, ghrelin inhibits the lipopolysaccharide (LPS)-induced release of pro-inflammatory cytokines from peripheral macrophages, T-cells and from LPS stimulated microglia. Here we sought to determine whether ghrelin attenuates pro-inflammatory cytokine release from dopaminergic neurones.

**Findings:**

The dopaminergic SN4741 cell-line, which derives from the mouse *substantia nigra* (SN) and expresses the ghrelin-receptor (growth hormone secretagogue receptor (GHS-R)) and the ghrelin-O-acyl transferase (GOAT) enzyme, was used to determine the neuro-immunomodulatory action of ghrelin. We induced innate immune activation via LPS challenge (1 μg/ml) of SN4741 neurones that had been pre-cultured in the presence or absence of ghrelin (1, 10, 100 nM) for 4 h. After 24 h supernatants were collected for detection of IL-1 beta (IL-1β ), TNF alpha (TNF-α) and IL-6 cytokines via enzyme linked immunosorbent assay (ELISA) analysis. Nuclear translocation of the transcription factor nuclear factor kappa B (NF-κB) was analyzed by Western blotting, and to determine viability of treatments a cell viability assay and caspase-3 immunohistochemistry were performed.

We provide evidence that while IL-1β and TNF-α were not detectable under any conditions, SN4741 neurones constitutively released IL-6 under basal conditions and treatment with LPS significantly increased IL-6 secretion. Pre-treatment of neurones with ghrelin attenuated LPS-mediated IL-6 release at 24 h, an affect that was inhibited by the GHS-R antagonist [D-Lys3]-GHRP-6. However, while ghrelin pre-treatment attenuated the LPS-mediated increase in NF-κB, there was no alteration in its nuclear translocation. Cell viability assay and caspase-3 immunocytochemistry demonstrated that the results were independent from activation of cytotoxic and/or apoptotic mechanisms in the neuronal population, respectively.

**Conclusion:**

Our results provide evidence that the gut-hormone, ghrelin, attenuates IL-6 secretion to LPS challenge in mid-brain dopaminergic neurones. These data suggest that ghrelin may protect against dopaminergic SN nerve cell damage or death via modulation of the innate immune response.

## Findings

Ghrelin is secreted from the stomach in response to nutrient restriction [1] and signals changes in metabolic status to the brain [2]. Circulating ghrelin crosses the blood–brain barrier (BBB) and binds to its receptor, growth hormone secretagogue receptor (GHS-R), in several brain regions, including the *substantia nigra* (SN) [3], where it modulates the activity and synaptic organization of midbrain dopamine neurones [4]. Of particular interest, the GHS-R has been shown to co-localize in the brain with the dopamine receptor 1 (D1R), and these receptors heterodimerize *in-vitro* to potentiate dopamine-induced cAMP accumulation [5]. In *in vitro* and rodent models of Parkinson’s disease (PD), acute ghrelin antagonizes 1-methyl-4-phenyl-1,2,3,6-tetrahydropyridine (MPTP)-induced toxicity in dopaminergic neurones of the mouse SN [6,7]. More recently, ghrelin and GHS-R knock-out animals displayed increased susceptibility to dopamine loss in the *substantia nigra pars compacta* (SNpc) following MPTP administration [8].

In addition to modulating dopamine function in mid-brain neurones, ghrelin has also been described as an anti-inflammatory hormone [9], inhibiting the release of pro-inflammatory cytokines in response to treatment with the bacterial endotoxin, lipopolysaccharide (LPS), in peripheral macrophages [10] and T-cells [11]; as well as from central nervous system (CNS) derived microglia [12]. Moreover, in the same study, exogenous ghrelin suppressed spinal cord levels of TNF-α, IL-1β and IL-6 mRNA in a mouse model of experimental autoimmune encephalomyelitis (EAE). As markers of inflammation have been observed in human PD [13] and peripheral [14] and striatal [15] LPS administration result in inflammation and degeneration of tyrosine hydroxylase (TH) immunopositive cells in the SNpc of adult rats, respectively, we sought to determine whether ghrelin’s neuromodulatory effect may involve attenuation of pro-inflammatory cytokine release from mid-brain dopaminergic neurones.

**Figure 1:**
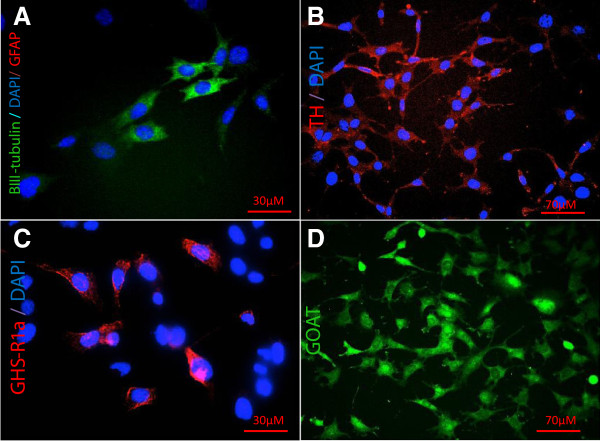
**Mouse mid-brain derived SN4741 cells express GHS-R, GOAT, BIII-tubulin and TH.** To determine whether SN4741 neurones were an appropriate cellular system to study ghrelin’s neuronal actions, we performed immunofluorescence (IF) to establish expression of key proteins. SN4741 cells were immunoreactive for βIII-tubulin **(A)**, TH **(B)**, GHS-R1a **(C)** and GOAT **(D)**. No immunoreactivity was observed for the glial marker, GFAP (A). For all negative controls the primary antibodies were excluded from the staining procedure. In addition, specificity of the GOAT antibody has been confirmed using an antibody-specific blocking peptide (Phoenix peptide cat.no 032–12; personal communication, Dr T Wells). The images are representative of three independent experiments performed with three replicates for each antigen.

First, we established the mouse mid-brain derived SN4741 cell-line [16] as an appropriate cellular system to study ghrelin’s neuronal actions. Cells (5 × 10^4^ per ml; Scepter, Millipore, Billerica, MA, USA) were cultured in Dulbecco’s modified Eagle’s medium (DMEM) (Gibco, Life Technologies, Paisley, UK) containing 10% fetal bovine serum (Gibco), 3% filtered glucose (Sigma, St Louis, MO, USA) and 1% L-Glutamine penicillin streptomycin (Sigma) at 37°C in air with 5% CO_2_, in 96-well plates (Corning, Tewksbury, MA, USA) for 24 h. Cells were fixed and processed according to the manufacturer’s instructions (NeuroTox-3, Millipore) prior to staining for the markers βIII-tubulin (rabbit polyclonal, Millipore, 1:100), GHS-R (goat polyclonal, Santa Cruz Biotechnology, Santa Cruz, CA, USA, 1:200), GOAT (rabbit polyclonal Phoenix Pharmaceuticals Inc, Burlingame, CA, USA, 1:500), GFAP (mouse monoclonal, Millipore, 1:100), TH (rabbit polyclonal, Abcam, Cambridge, UK, 1:500), and the cytotoxic marker, caspase-3 (rabbit polyclonal, Promega, Southampton, UK, 1:250) for 1 h at room temperature. Followed by incubation with appropriate secondary antibody FITC-donkey anti-rabbit (Millipore, 1:200), Cy3-donkey anti-mouse (Millipore, 1:200), AF-568-donkey anti-goat (Life Technologies, Paisley, UK, 1:200) and Hoechst nuclear stain (5 μg/ml) for 1 h at room temperature protected from light. Fluorescent images, captured using a x20 objective on an In Cell Analyser 2000 (GE Healthcare, Cardiff, UK), demonstrated immunoreactivity for the neuronal marker, βIII-tubulin (Figure 1A); the rate-limiting enzyme for dopamine synthesis, TH (Figure 1B); the ghrelin receptor, GHS-R (Figure 1C); and the enzyme for ghrelin acylation, GOAT (Figure 1D); thus confirming the suitability of this cell line to investigate ghrelin function. No immunoreactivity was observed for the glial marker, GFAP (Millipore) (Figure 1A).

**Figure 2:**
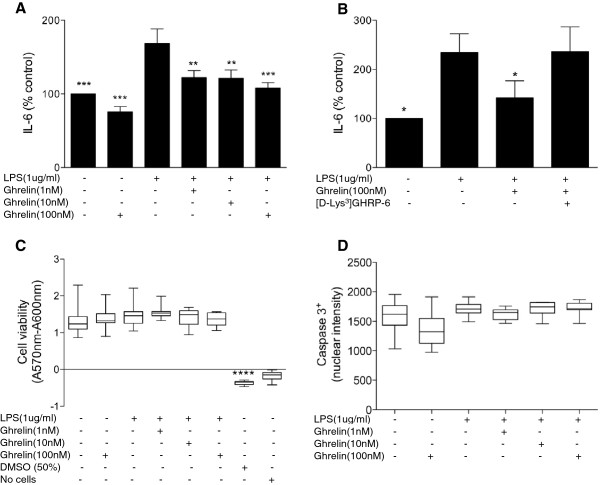
**Ghrelin inhibits LPS-induced IL-6 release in a GHS-R dependent manner.** To determine whether ghrelin modulates the secretion of inflammatory cytokines in mid-brain neurones, we induced innate-immune activation via LPS (1 μg/ml). Neurones (3 to 5 x 10^5^ per ml) were treated with ghrelin (1, 10, 100 nM) for 4 h prior to LPS (1 μg/ml) challenge; subsequently, supernatants were collected 24 h later for detection of IL-6 via ELISA. **A)** Pre-treatment with ghrelin at 1, 10, and 100 nM significantly reduced LPS-induced IL-6 secretion. Data are normalized to cell number and expressed as a percentage of the control value (mean ± s.e.m), which was 774.8 ± 306.7 pg/ml, and represent four independent experiments performed in-triplicate for each concentration point. **B)** Pre-incubation of ghrelin with the ghrelin-receptor antagonist, [D-Lys-3]-GHRP-6, blocked ghrelin’s inhibitory effect on LPS-induced IL-6 secretion. The data **(**expressed as in **A)** with a control value of 815.8 ± 319.8 pg/ml represent the mean ± s.e.m of five independent experiments performed with four replicates for each concentration point. To ensure that treatments were not inducing cytotoxic or apoptotic changes in SN4741 cells, we tested cell viability (CellTiter Blue, Promega) **(C)** and caspase-3 immunoreactivity **(D)**. The CellTiter-Blue® Assay, which is based on the ability of living cells to convert a redox dye (resazurin; Absorbance^max^ = 605 nm) into a fluorescent end product (resorufin; Absorbance^max^ = 573 nm) (viable cells reduce resazurin into resorufin) demonstrated that ghrelin and LPS had no significant effect on viability **(C)**. Similarly, nuclear intensity of caspase-3 was not significantly altered by treatments **(D)**. The data represent the mean ± SEM of two independent experiments with seven replicates for each concentration point. Statistical analysis was performed using one-way ANOVA with Dunnett’s multiple comparison. *P* <0.05 regarded as significant (*P <0.05; ***P <0.001; ****P <0.0001 vs LPS).

To determine whether SN4741 neurones constitutively release inflammatory cytokines and whether ghrelin modulates their secretion in response to endotoxin challenge, cells (2 to 5 × 10^4^ per ml) were grown in 24-well plates, cultured as before and treated with acyl-ghrelin (Phoenix Pharmaceuticals cat.no. 031–31: 1, 10, 100 nM) for 4 h prior to LPS (1 μg/ml, Ultrapure LPS *E. coli* 0111:B4, Invivogen, San Diego, CA, USA) challenge. This dose of LPS is consistent with previously published work [8]; moreover, higher doses (2 μg and 10 μg) did not further augment IL-6 secretion in SN4741 cells (data not shown). Supernatants were collected 24 h later for detection of the pro-inflammatory cytokines TNF-α, IL1-β and IL-6 via ELISA (DuoSet, R&D Systems, Abingdon, UK) according to the manufacturer’s instructions. While TNF-α and IL1-β were not detectable under any conditions, SN4741 neurones constitutively released IL-6 and augmented its release in response to LPS (Figure 2A). This supports previous data suggesting that neurones are capable of mediating inherent cytokine responses via the synthesis and secretion of IL-6 [17]. Pre-treatment of neurones with ghrelin resulted in the significant attenuation of LPS-mediated IL-6 release (Figure 2A). Subsequently, to establish whether ghrelin inhibits LPS-induced IL-6 release via activation of its receptor, GHS-R, cells were pre-incubated in the presence of the selective GHS-R antagonist, [D-Lys3]-GHRP-6 (1,000 nM). Subsequent collection of supernatant and ELISA for IL-6 showed that ghrelin-mediated inhibition of LPS-induced IL-6 was prevented by the presence of [D-Lys3]–GHRP-6 (Figure 2B). To determine whether ghrelin (100 nM) and LPS treatment (as above) regulated gene expression of IL-6 and toll-like receptor 4 (TLR4), total RNA was extracted (Norgen Biotek, Thorold, ON, Canada; cat. no. 23000, according to the manufacturer’s instructions) from SN4741 cell lysates (n = 3 per group, from two independent experiments) at the 24 h time point. Subsequent RT-qPCR and melt-curve analysis confirmed the expression of IL-6 and TLR4 in SN4741 cells (mouse IL-6 forward primer TCCATCCAGTTGCCTTCTTG, reverse primer GGTCTGTTGGGAGTGGTATC; mouse TLR4 forward primer CTGGCTAGGACTCTGATCATG, reverse primer GCATTGGTAGGTAATATTAGGAACTA; Primer Design, Southampton, UK). Using the ΔΔCt method, quantification of IL-6 and TLR4 with individual samples normalized to β-actin expression (Primer Design), which demonstrated invariant expression between samples, showed that IL-6 and TLR4 mRNA levels were not altered (that is, greater than a two-fold change relative to control) by ghrelin or ghrelin/LPS co-treatments. However, LPS treatment alone induced a 2.03-fold and 2.76-fold reduction in the level of IL-6 and TLR4 mRNA, respectively. This may represent a negative feedback response to limit the potential detrimental consequences of excess IL-6 and TLR4 signaling in these neurones and is consistent with the phenomenon of LPS-tolerance observed in peritoneal macrophages [18].

To ensure culture treatments were not inducing cytotoxic changes in the SN4741 neurones, cell viability was assessed using the CellTiter Blue assay (Promega) performed according to the manufacturer’s instructions. Briefly, cells were cultured with ghrelin followed by LPS stimulation prior to incubation with CellTiter-Blue reagent for 13 h at 37°C. Absorbance readings at 570 to 600 nm using POLARstar Omega plate Reader (BMG Labtech, Aylesbury, UK) showed that culture treatments did not induce cytotoxic changes (Figure 2C). Furthermore, there was no alteration in the nuclear intensity of the apoptotic marker, caspase-3, in SN4741 cells following the same treatments (Figure 2D). The presence of nuclear caspase-3, which is dependent on its proteolytic activation [19], was detected by immunofluorescence and quantified using In Cell Analyser Developer™ software (GE Healthcare). Briefly, nuclear intensity images were acquired using a X10 objective and a watershed segmentation algorithm was used to distinguish individual nuclei; detected binary objects less than 40 μm^2^ and greater than 200 μm^2^ were subsequently removed from the analysis to reduce false and double counting of viable nuclei respectively.

Finally, protein levels of the transcription factor NF-κB, previously implicated in the ghrelin-mediated inhibition of cytokine release in human endothelial cells [20] and murine macrophages [10], were analyzed by Western blot in nuclear and cytoplasmic cell extracts. SN4741 cells were seeded at 4 × 10^4^ per ml and grown under normal conditions for 24 h prior to overnight incubation in serum free media. Cells were pre-treated in the presence or absence of acyl-ghrelin (100 nM) or [D-Lys3]-GHRP-6 (1,000 nM) for 4 h prior to LPS (1 μg/ml) stimulation for 2 h.

**Figure 3:**
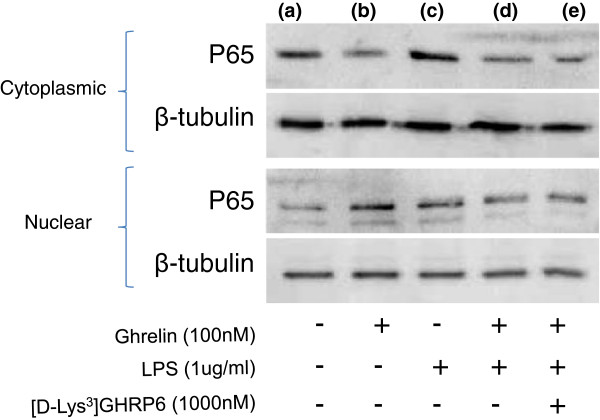
**Nuclear translocation of transcription factor NF-κB is not associated with ghrelin-mediated inhibition of IL-6 release.** NF-κB has been implicated in ghrelin-mediated inhibition of cytokine release in murine macrophages. To determine if a similar mechanism exists in SN4741 neurones Western blots were performed on cytoplasmic and nuclear cell extracts following treatments. Under basal conditions p65 was abundant in the cytoplasmic extract **(a)**, whereas ghrelin treatment seemingly promoted shuttling of p65 into the nuclear compartment **(b)**. However, while LPS treatment alone elevated p65 **(c)** and ghrelin/[D-Lys3]-GHRP-6 treatments attenuated p65 **(d, e)**, there was no apparent alteration in cytoplasmic to nuclear ratio to indicate regulation via p65. The blots are representative of two independent experiments.

Cytoplasmic and nuclear proteins were extracted (Nuclear Extract Kit, Active Motif, Carlsbad, CA, USA) and protein concentration determined by DC Protein Assay (Bio-Rad Laboratories, Hertfordshire, UK). Lysates (5 μg) were mixed with Laemmli Lysis Buffer (Sigma, St. Louis, MO, USA) in a 1:1 ratio and heated at 95°C to denature proteins. Samples were briefly spun down and proteins separated using 12% SDS-PAGE (Expedeon, Cambridgeshire, UK) gel electrophoresis and transferred to a PVDF membrane at 200 V for 75 minutes. Membranes were blocked with 3% milk (in TBST) for 60 minutes at room temperature with mild agitation to reduce non-specific binding then probed for the p65 subunit of the NF-ĸB complex (rabbit polyclonal, Santa Cruz Biotechnology, sc-372, used at 1:1,000) and the loading control β-tubulin (rabbit polyclonal, Abcam, Cambridge, UK, ab6046, used at 1:500) for 90 minutes at room temperature with mild agitation. Subsequently, membranes were incubated with horseradish peroxidase-conjugated secondary antibody (goat polyclonal to rabbit IgG, Abcam, ab6721, used at 1:2,000) for 45 minutes at room temperature with mild agitation. Immunoreactive protein bands were visualized using the enhanced chemiluminescence system (Immun-star WesternC Chemiluminescent kit, Bio-Rad Laboratories, Hertfordshire, UK) after a 15-second exposure and protein content was normalized to the loading control, β-tubulin. Under basal conditions p65 was abundant in the cytoplasmic extract (Figure 3a), whereas ghrelin treatment seemingly promoted shuttling of p65 into the nuclear compartment (Figure 3b). However, while LPS treatment alone elevated p65 in both cytoplasmic and nuclear compartments (Figure 3c), and pre-treatment with ghrelin and [D-Lys3]-GHRP-6 attenuated p65 (Figure 3d, e), there was no apparent alteration in the cytoplasmic to nuclear ratio to indicate regulation via p65. This suggests that ghrelin’s inhibitory effect on IL-6 release from mid-brain dopamine neurones may not be associated with NF-κB nuclear translocation. Given previous findings [10,20] we cannot rule out the possibility that 1 μg/ml LPS represents a sub-maximal dose for NF-κB nuclear translocation or that such translocation is temporally regulated. Alternatively, the NF-κB response to LPS was analyzed in midbrain neurones in the absence of astrocytes and may, therefore, indicate an alternative mechanism of IL-6 regulation for this cell population. Another possibility, consistent with unaltered levels of IL-6 gene expression, is that the observed inhibitory action of ghrelin may be mediated by modulating secretory vesicle physiology rather than transcriptional mechanisms.

## Conclusion

Given that circulating ghrelin crosses the BBB, binds to its receptor in the SNpc [3] and protects against dopamine nerve cell death in toxin-models of PD [6,7], we suggest that ghrelin is an endogenous neuro-active hormone that plays a key role in the brain’s adaptation to nutrient supply. We provide evidence that ghrelin’s effect on dopamine mid-brain neurones may be mediated, at least in part, via directly modulating IL-6 release. These data are consistent with ghrelin protecting against dopamine neurone death via inhibition of microglial activation [21] and suggest that ghrelin may confer protection via the modulation of neurone-glia communication [22]. Moreover, as circulating post-prandial ghrelin is significantly reduced in PD patients [23] and cerebrospinal fluid levels of IL-6 are increased in both juvenile and adult onset PD [24,25], we speculate that a reduction in circulating ghrelin may exacerbate dopaminergic cell loss in human PD due to elevated IL-6 acting as a primer for neurodegeneration. However, as IL-6 is a pleiotropic cytokine with mitogenic and neurotrophic actions, the potential for ghrelin protecting against dopaminergic SN nerve cell damage or death via attenuated IL-6 requires further *in-vivo* elucidation.

In conclusion, we describe a previously uncharacterized innate-immune response within mesencephalic neurones whereby an endogenous brain-penetrant hormone that is regulated by feeding, inhibits neuronal cytokine secretion in response to immune-challenge.

## Abbreviations

BBB: blood–brain barrier; cAMP: cyclic adenosine monophosphate; CNS: central nervous system; D1R: dopamine receptor 1; DMEM: Dulbecco’s modified Eagle’s medium; EAE: experimental autoimmune encephalomyelitis; ELISA: enzyme linked immunosorbent assays; FITC: fluorescein isothiocyanate; GFAP: glial fibrillary acidic protein; GHS-R: growth hormone secretagogue receptor; GOAT: ghrelin-O-acyl transferase; IL-6: interleukin-6; LPS: lipopolysaccharide; MPTP: 1-methyl-4-phenyl-1,2,3,6-tetrahydropyridine; mRNA: messenger ribonucleic acid; NF-kB: nuclear factor kappa beta; PD: Parkinson’s disease; RT-qPCR: reverse transcription quantitative polymerase chain reactive; SN: substantia nigra; SNpc: substantia nigra pars compacta; TH: tyrosine hydroxylase; TLR4: toll-like receptor; TNF-a: tumor necrosis factor-alpha.

## Competing interest

All authors declare that they have no competing interests.

## Authors’ contributions

JSD conceived the study. JSD, IMS and MIR designed the experiments. ALB, RW and JSD performed the experiments. MRB, ALB and JSD analyzed the data and JSD wrote the manuscript. All authors read and approved the final manuscript.
